# MEPE is a novel regulator of growth plate cartilage mineralization

**DOI:** 10.1016/j.bone.2012.06.022

**Published:** 2012-09

**Authors:** K.A. Staines, N.C.W. Mackenzie, C.E. Clarkin, L. Zelenchuk, P.S. Rowe, V.E. MacRae, C. Farquharson

**Affiliations:** a The Roslin Institute and Royal (Dick) School of Veterinary Studies, The University of Edinburgh, Easter Bush, Midlothian EH25 9RG, UK; b Centre for Biological Sciences, University of Southampton, Southampton General Hospital, Southampton SO16 6YD, UK; c Department of Internal Medicine, The Kidney Institute and Division of Nephrology, University of Kansas Medical Center, Kansas City, KS, USA

**Keywords:** MEPE, ASARM, Growth plate, Mineralization, Chondrocyte

## Abstract

Matrix extracellular phosphoglycoprotein (MEPE) belongs to the SIBLING protein family which play key roles in biomineralization. Although the growth plates of MEPE-overexpressing mice display severe morphological disruption, the expression and function of MEPE in growth plate matrix mineralization remains largely undefined. Here we show MEPE and its cleavage product, the acidic serine aspartate-rich MEPE-associated motif (ASARM) peptide, to be localised to the hypertrophic zone of the growth plate. We also demonstrate that the phosphorylated (p)ASARM peptide inhibits ATDC5 chondrocyte matrix mineralization. Stable MEPE-overexpressing ATDC5 cells also had significantly reduced matrix mineralization in comparison to the control cells. Interestingly, we show that the addition of the non-phosphorylated (np)ASARM peptide promoted mineralization in the ATDC5 cells. The peptides and the overexpression of MEPE did not affect the differentiation of the ATDC5 cells. For a more physiologically relevant model, we utilized the metatarsal organ culture model. We show the pASARM peptide to inhibit mineralization at two stages of development, as shown by histological and μCT analysis. Like in the ATDC5 cells, the peptides did not affect the differentiation of the metatarsals indicating that the effects seen on mineralization are direct, as is additionally confirmed by no change in alkaline phosphatase activity or mRNA expression. In the metatarsal organ cultures, the pASARM peptide also reduced endothelial cell markers and vascular endothelial growth factor mRNA expression. Taken together these results show MEPE to be an important regulator of growth plate chondrocyte matrix mineralization through its cleavage to an ASARM peptide.

## Highlights

► MEPE and its ASARM peptide are localised to the hypertrophic chondrocytes of the growth plate. ► Phosphorylated ASARM peptides inhibit chondrocyte matrix mineralisation in ATDC5 cells and in metatarsal organ cultures. ► This inhibition is independent of altered alkaline phosphatase activity. ► Phosphorylated ASARM peptides inhibited the mRNA expression of endothelial cell specific markers and pro-angiogenic VEGF.

## Introduction

Linear bone growth involves the replacement of a cartilaginous template by mineralized bone through endochondral ossification. This growth process is orchestrated by various actions at the growth plate, a developmental region consisting of chondrocytes in distinct cellular zones. The proliferation, hypertrophy and apoptosis of these growth plate chondrocytes are regulated by a tight array of factors ensuring effective cartilage mineralization and thus longitudinal growth [1].

Hydroxyapatite (HA) crystals form associated with the trilaminar membrane bound matrix vesicles (MV) which in the growth plate are localised to the mineralized longitudinal septae and form from the plasma membrane of the terminal hypertrophic chondrocytes [2]. Mineralization is a biphasic process which is under tight control so as to ensure levels of calcium (Ca^2 +^) and inorganic phosphate (P_i_) are permissive for effective HA formation [2]. Three molecules have been identified as imperative in controlling levels of the mineralization inhibitors inorganic pyrophosphate (PP_i_), and osteopontin [2,3]. These are alkaline phosphatase (ALP), a nucleotide pyrophosphatase/phosphodiesterase isozyme (NPP1), and the Ankylosis protein (ANK). However, mechanisms beyond the supply and hydrolysis of PP_i_ likely exist to control chondrocyte matrix mineralization.

Once such mechanism could involve matrix extracellular phosphoglycoprotein (MEPE, OF45). This was originally isolated and cloned from tumors of oncogenic hypophosphatemic osteomalacia (OHO) as a candidate substrate for phosphate-regulating gene with homologies to endopeptidases on the X chromosome (PHEX) [4]. MEPE is a 56–58 kDa SIBLING (small integrin-binding ligand N-linked glycosylated) protein along with dentin matrix protein 1 (DMP1), osteopontin (OPN), dentin sialophosphoprotein (DSPP) and bone sialoprotein (BSP) [5]. SIBLING proteins are expressed in bone and dentin, and have roles in extracellular matrix (ECM) formation and mineralization [6]. Their structures are similar; all display an Arg-Gly-Asp (RGD) motif which facilitates cell attachment, and all are commonly located on the human chromosome 4q21-23 [4,7,8].

In bone, MEPE is primarily expressed by osteocytes, but *Mepe* mRNA expression has also been observed in osteoblasts [9]. The expression of MEPE is increased during osteoblast matrix mineralization suggesting a function for MEPE in bone mineralization [10,11]. This has been further fuelled by analysis of the MEPE null mouse in which the ablation of MEPE leads to an increased bone mass due to increased numbers and activity of osteoblasts [12]. Furthermore, the overexpression of MEPE in mice, under the control of the Col1a1 promoter, leads to defective mineralization coupled with an increased level of MEPE-ASARM peptides in bone [13]. The MEPE-overexpressing mice displayed wider epiphyseal growth plates, with associated expanded primary spongiosa and a significant decrease in mineral apposition rate [13]. Further studies in vitro have confirmed the inhibitory effect of MEPE on mineralization and have identified that MEPE is cleaved to a 2.2 kDa ASARM peptide which causes this effect [14,15]. The ASARM motif is located immediately downstream of a cathepsin B cleavage site, and it is responsible for the mineralization defect observed in X-linked hypophosphatemic rickets, the most common form of inherited rickets [4,14,15]. This defect can be reversed by administration of cathepsin inhibitors CAO74 or pepstatin [16]. PHEX plays a central role in the protection of MEPE from proteolytic cleavage by cathepsin B; it can bind to MEPE and prevent the release of the ASARM peptide [17]. The *Hyp* mouse, a spontaneous *Phex* knockout model, has an increased expression of cathepsin D, an upstream activator of cathepsin B [16]. Therefore PHEX may also assist in decreasing the activation of cathepsin B.

Previous studies have shown that the post translational modification of the MEPE-ASARM peptide is key to its functional role. MEPE has a number of potential casein kinase II phosphorylation motifs, and it is here that the ASARM peptide is phosphorylated at 3 serine residues [4]. This has been shown to inhibit mineralization in murine calvarial osteoblasts and in bone marrow stromal cells by the direct binding of the MEPE-ASARM peptide to HA crystals [14,18].

To elucidate the interactions of MEPE in the growth plate, this study was undertaken to examine the presence and function of MEPE and its ASARM peptide in growth plate matrix mineralization during the endochondral ossification process. The data indicated that MEPE is expressed by growth plate chondrocytes, in particular in the hypertrophic zone of chondrocytes consistent with a potential role in matrix mineralization. MEPE has a functional role in the inhibition of chondrocyte ECM mineralization, involving its cleavage, and subsequent phosphorylation, to the ASARM peptide.

## Materials and methods

### Animals

Proximal tibiae from 3‐ and 4‐week‐old C57/BL6 mice were dissected and excess tissue was removed before preparation of the tissues for in situ hybridization, immunohistochemistry and microdissection of the growth plate. For metatarsal organ culture, the middle three metatarsals were aseptically dissected from E17 and E15 C57/BL6 mice. All experimental protocols were approved by Roslin Institute's Animal Users Committee and the animals were maintained in accordance with UK Home Office guidelines for the care and use of laboratory animals.

### In situ hybridization

Bone tissue was fixed in 10% neutral buffered formalin (Sigma, Gillingham, UK) for 48 h at 4 °C, before being decalcified in 10% ethylenediaminetetraacetic acid (EDTA) (Sigma) pH 7.4 at 4 °C for approximately 4 weeks with regular changes. Tissues were dehydrated and embedded in paraffin wax using standard procedures, before being sectioned at 5 μm. A full length murine MEPE cDNA IMAGE clone (ID: 8733911) was purchased (Source BioScience UK Ltd, Nottingham). Anti-sense and sense constructs were linearised, using *Nco1*, and digoxigenin-labeled cRNA probes were synthesised using T3 and T7 RNA polymerases respectively (Roche, Burgess Hill, UK). Hybridizations were completed following an optimised in situ hybridization protocol as previously detailed [19].

### Growth plate microdissection

Bone tissue samples were coated in 5% polyvinyl acetate and then immersed in a cooled hexane bath for 30 s after which they were stored at − 80 °C until use. Using optimal cutting temperature (OCT) embedding medium (Brights, Huntingdon, UK) 30 μm sections were cut at − 30 °C (Brights, OT model cryostat), and then stored at − 80 °C. Slides were briefly thawed and then microdissection was performed as previously detailed [20]. For each zone, tissue was dissected from both proximal tibias of three animals (14–22 sections) and RNA isolation was performed as previously described [21].

### Immunohistochemistry

After dissection, tissue was fixed in 70% ethanol for 24 h at 4 °C before being decalcified in 10% EDTA (pH 7.4) for approximately 4 weeks at 4 °C with regular changes. Tissues were finally dehydrated and embedded in paraffin wax, using standard procedures, after which they were sectioned at 5 μm. For immunohistochemical analysis, sections were dewaxed in xylene and rehydrated. Sections were incubated at 37 °C for 30 min in 0.1% trypsin (Sigma) for antigen demasking. Endogenous peroxidases were blocked by treatment with 0.03% H_2_O_2_ in methanol (Sigma). From this point onwards, the Vectastain ABC (Goat) kit (Vector Laboratories, Peterborough) was used according to the manufacturer's instructions. ASARM and MEPE primary antibodies were used at a dilution of 1/200 with rabbit IgG used as a control [13]. Cathepsin B primary antibodies (R&D Systems, Abingdon, UK) were used at a dilution of 2 μg/ml with goat IgG used as an appropriate control. The sections were dehydrated, counterstained with haematoxylin and mounted in DePeX.

### MEPE‐ASARM peptides

MEPE-ASARM peptides were synthesised (Peptide Synthetics, UK) as phosphorylated ASARM (pASARM) with the sequence RDDSSESSDSG(Sp)S(Sp)SSE(Sp)SDGD, and non-phosphorylated ASARM (npASARM) with the sequence RDDSSESSDSGSSSESDGD. pASARM and npASARM peptides were added to ATDC5 cells and metatarsal organ cultures at concentrations of 10, 20 and 50 μM, with controls treated with a DMSO (Sigma) carrier only. In further studies, peptides were added at a final concentration of 20 μM with experiments being performed at least 3 times.

### Metatarsal organ culture

Embryonic metatarsal organ cultures provide a well‐established model of endochondral bone growth [22–24]. Metatarsal bones were cultured in a humidified atmosphere (37 °C, 5% CO_2_) in 24-well plates for up to 10 days. Each culture well contained 300 μl *α*-minimum essential medium (MEM) supplemented with 0.2% BSA Fraction V; 1 mmol/l β-glycerophosphate (βGP); 0.05 mg/ml L-ascorbic acid phosphate; 0.05 mg/ml gentamicin and 1.25 μg/ml fungizone (Invitrogen, Paisley, UK) as previously described [22]. For the E17 bones, the medium was changed every second or third day and for the E15 bones, the medium was not changed throughout the culture period [25]. Concentrations of peptide and DMSO carrier were however added every second day.

### Morphometric analysis of metatarsals

The total length of the bone through the centre of the mineralizing zone was determined using image analysis software (DS Camera Control Unit DS-L1; Nikon) every second or third day. The length of the central mineralization zone was also measured. All results are expressed as a percentage change from harvesting length which was regarded as baseline.

### 3D-Microtomography of metatarsals

Metatarsals were fixed in 70% ethanol, stained with eosin dye (for visualisation) and then embedded in paraffin blocks. Samples were then were scanned with a high-resolution μCT (μCT40; Scanco Medical, Southeastern, PA) as previously described [13,16]. Data were acquired at 55 KeV with 6 μm cubic voxels. Three-dimensional reconstructions for bone samples were generated with the following parameters: Gauss Sigma = 4.0; Support = 2, Lower Threshold = 90 and Upper Threshold = 1000. Tissue mineral density was derived from the linear attenuation coefficient of threshold bone through precalibration of the apparatus for the acquisition voltage chosen. The bone volume (BV/TV) was measured using sections encompassing the entire metatarsal on a set of 85 sections that was geometrically aligned for each sample.

### Metatarsal [^3^H]-thymidine proliferation assay

On day 7 of culture, 3 μCi/ml [^3^H]-thymidine (Amersham Biosciences, Little Chalfont, UK) was added to each metatarsal for the last 6 h of culture [22]. After washing in PBS, the unbound thymidine was extracted using 5% trichloroacetic acid (Sigma). Metatarsals were then washed in PBS before being solubilised (NCS-II tissue solubiliser, 0.5 N, Amersham) at 60 °C for 1 h. [^3^H]-thymidine incorporated into DNA was determined using a scintillation counter.

### Cell culture

Chondrogenic ATDC5 cells (RIKEN cell bank, Ibaraki, Japan) were utilized as a well-established model of chondrocyte matrix mineralization with previous studies detailing their chondrogenic differentiation and subsequent mineralization [26]. Cells were cultured in differentiation medium (DMEM/F-12 (1:1) with GlutaMAX I containing 5% FBS, 1% insulin transferrin and selenium, 1% sodium pyruvate and 0.5% gentamicin (Invitrogen)) at a density of 6000 cells/cm^2^. 10 mM beta-glycerophosphate (βGP) and 50 μg/ml ascorbic acid were added once the cells had reached confluency. Cells were incubated in a humidified atmosphere (37 °C, 5% CO_2_) for up to 15 days with medium changed every second or third day.

### Plasmid construction

The full length murine MEPE cDNA (IMAGE clone ID: 8733911) was supplied within a pCR4.TOPO vector (Source BioScience UK Ltd, Nottingham). The cDNA sequence was excised by digestion with *EcoRI* and sub-cloned into the pEN.Tmcs (MBA-251; LGC Standards, Middlesex, UK) using T4 DNA ligase (Roche). The expression vector pLZ2-Ub-GFP (kind gift from D. Zhao, Roslin Institute) was digested with *BamHI* and *XbaI* to remove the GFP cDNA. The MEPE cDNA was excised from the pEN.T-MEPE sub-cloning vector using *BamHI* and *XbaI* and ligated into pLZ2-Ub backbone to create a Ubiquitin driven MEPE expression construct, pLZ2-Ub.MEPE. To create the empty vector control (pLZ2-Ub.EMPTY) the pLZ2-Ub backbone was blunted using T4 polymerase (New England Bioscience, Hitchin, UK) and re-ligated.

### Establishment of stable MEPE-overexpressing ATDC5 cells

ATDC5 cells were maintained in differentiation medium as previously described and seeded at 150,000 cells/cm^2^. Cells were transfected with pLZ2-Ub.MEPE and pLZ2-Ub.EMPTY constructs at a ratio of 7:2 FuGENE HD (Roche) to DNA, according to the manufacturer's instructions. Blasticidin resistant colonies were picked using cloning cylinders (Sigma), expanded, frozen and maintained at − 150 °C until further use. Three MEPE-overexpressing and three empty vector clones were picked for analysis.

### Real-time quantitative PCR (RT-qPCR)

RNA was extracted from ATDC5 cell cultures using an RNeasy mini kit (Invitrogen) according to the manufacturer's instructions. For metatarsal organ cultures, 4 bones from each control or experimental group were pooled in 100 μl Trizol reagent (Invitrogen) at days 5 and 7 of culture, and RNA was extracted according to the manufacturer's instructions. For each sample, total RNA content was assessed by absorbance at 260 nm and purity by A260/A280 ratios, and then reverse-transcribed. RT-qPCR was performed using the SYBR green detection method on a Stratagene Mx3000P real-time qPCR system (Stratagene, CA, USA), or a LC480 instrument (Roche). Primers were purchased (PrimerDesign Ltd, Southampton, UK) or designed in house and synthesised by MWG Eurofins, London, UK, or Sigma. Sequences are detailed in Supplemental Table S1. Reactions were run in triplicate and routinely normalized against *18S* or *β-actin*.

### Endpoint PCR analysis

Expression of specific pro-angiogenic vascular endothelial growth factor (VEGF)-A isoforms namely VEGF_120,164_ and _188_ was analysed as previously detailed [27]. The VEGF isoform primer sequences were: forward GAAGTCCCATGAAGTGATCCAG and reverse TCACCGCCTTGGCTTGTCA. Located on exon 3 (forward) and exon 8 (reverse), these amplify all the isoforms of murine VEGF. Different isoform mRNA expression profiles were identified in a 2.5% agarose (Sigma) gel according to the molecular weight of PCR products using cDNA synthesised from equal amounts of RNA. Product band densities were analysed using Image J software (U. S. National Institutes of Health, Maryland, USA).

### Histological procedures

After 15 days of culture, calcium and collagen deposition in ATDC5 cells were evaluated by alizarin red stain (Sigma) and sirius red stain (Biocolor Ltd., Newtownabbey, UK) respectively [28]. Cells were fixed in 4% paraformaldehyde following washes with PBS. 2% alizarin red (pH 4.2) was added to the cell layers for 5 min at room temperature and then rinsed off with distilled water. Alizarin red-stained cultures were extracted with 10% cetylpyridinium chloride for 10 min [28–30]. Sirius red was added to cell cultures for 1 h at room temperature before being rinsed with distilled water. 0.001 M hydrochloric acid was then used to remove unbound dye. To quantify staining, 0.1 M sodium hydroxide was used for 30 min. The optical density (OD) of the alizarin red and sirius red digests was measured at 570 nm by spectrophotometry (Multiskan Ascent, Thermo Electron Corporation, Vantaa, Finland). Proteoglycan synthesis content was evaluated by staining the cell layers with alcian blue (Sigma). Cells were fixed in 95% methanol for 20 min and stained with 1% alcian blue 8GX in 0.1 M HCl overnight. Alcian blue-stained cultures were extracted with 1 ml of 6 M guanidine–HCl for 6 h at room temperature and the OD was determined at 630 nm by spectrophotometry [28].

### Alkaline phosphatase enzyme activity

At the end of the culture period, alkaline phosphatase (ALP) activity within the metatarsal bones was determined using an assay for ALP (Thermo Fisher Scientific, Epsom, UK) according to the manufacturer's instructions. Briefly, each metatarsal was permeabilized in 100 μl of 10 mmol/l glycine (pH 10.5) containing 0.1 mmol/l MgCl_2_, 0.01 mmol/l ZnCl_2_, and 0.1% Triton X-100 by freeze-thawing three times [22]. Each extract was assayed for ALP activity by measuring the rate of cleavage of 10 mM *p*-nitrophenyl phosphate. Total ALP activity was expressed as nanomoles *p*-nitrophenyl phosphate hydrolysed per minute per bone.

### Lactate dehydrogenase activity

Lactate dehydrogenase (LDH) activity was determined in the culture medium of 15-day-old 0 mM and 10 mM βGP treated ATDC5 cells using a kit from Roche Diagnostics (Lewes, East Sussex, UK). LDH activity was related to the total LDH activity of the cultures.

### Statistical analysis

Data were analysed by one-way analysis of variance (ANOVA), the Student's *t*-test, or a suitable non-parametric test using Sigma Plot 11 (Germany). All data are expressed as the mean ± SEM.

## Results

### The expression of MEPE in the murine growth plate

**Fig. 1:**
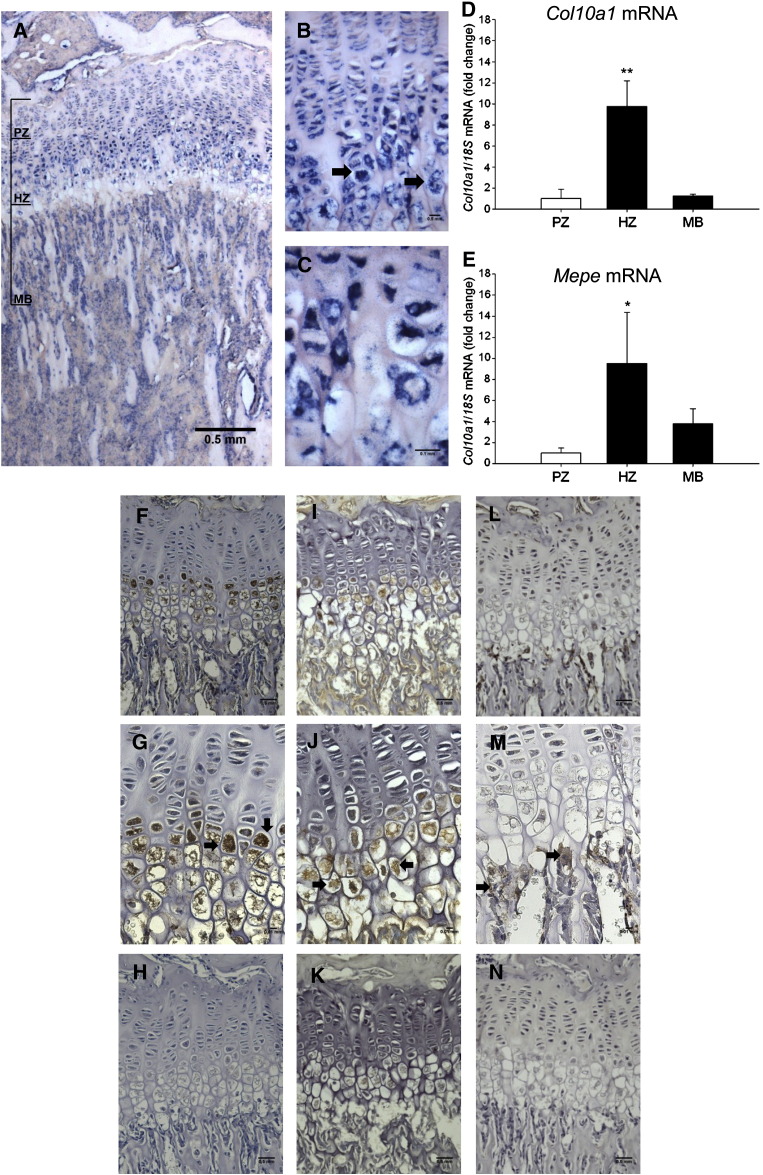
In situ hybridization of *Mepe* in 3-week-old mouse tibia. *Mepe* was found to be abundantly expressed by growth plate chondrocytes and osteoblasts of the metaphysis (A). Expression was present in both the proliferating zone (PZ) and the hypertrophic zone (HZ) of the growth plate, as indicated by the arrows (B), with an apparent increase in expression in the hypertrophic zone of chondrocytes (C). Microdissection of the growth plate was adopted to assess *Mepe* mRNA expression. The accuracy of the microdissection technique was determined by the relative change in *col10a1* mRNA expression throughout the zones of the growth plate and the trabecular bone (D). This was then used to examine the relative change in *Mepe* mRNA expression in these zones (E). Immunohistochemistry shows MEPE (F and G) and the MEPE-ASARM peptide (I and J) to be expressed in the tibia of 4-week-old growth plates. Its expression in the growth plate is limited to the hypertrophic zone (HZ) of chondrocytes, as indicated by the arrows. Cathepsin B immunolocalization (L and M) was exclusive to the chondro-osseous junction, as highlighted by the arrows. Representative images of appropriate negative control are shown (H, K, and N). Values generated by RT-qPCR and normalized to *18S*. Data are represented as mean ± SEM, in comparison to the PZ, *P < 0.05 **P < 0.005. Scale bars are (A, B, F, H, I, K, L, N) 0.5 mm, (C) 0.1 mm, and (G, J, M) 0.01 mm.

To assess the expression of MEPE by growth plate chondrocytes we examined *Mepe* mRNA localization in the murine growth plate of 3-week-old mice by in situ hybridization. *Mepe* was expressed abundantly by growth plate chondrocytes and by osteoblasts within the metaphysis (Fig. 1A). In the growth plate, high levels of *Mepe* mRNA were observed, especially in the hypertrophic chondrocytes (Fig. 1B and C). This spatial expression pattern was further examined and quantified by microdissection of growth plates. To validate the microdissection technique, RT-qPCR of collagen type X mRNA expression was conducted to ensure that the hypertrophic zone could be considered as an enriched pool of hypertrophic chondrocytes (Fig. 1D). There was approximately a 10-fold increase in collagen type X mRNA expression in the hypertrophic zone in comparison to the proliferative zone (*P* < 0.001). This is in concordance with previous studies done using a similar technique [31]. *Mepe* mRNA had a significantly higher expression (*P* < 0.05) in the hypertrophic zone in comparison to the proliferative zone of the growth plate (Fig. 1E). Immunolocalization of MEPE and the MEPE-ASARM peptide in 4-week-old growth plates verified the in situ hybridization and microdissection data as demonstrated by its localization to the hypertrophic zone of chondrocytes (Fig. 1F and H). This ASARM peptide is cleaved from MEPE by cathepsin B; thus, we examined the immunolocalization of cathepsin B in the growth plate (Fig. 1J). Here we show it to be expressed at the chondro-osseous junction as is in concordance with previous studies [32,33]. Representative images of the appropriate negative controls are shown (Fig. 1G, I and K). Together these data indicate that MEPE-ASARM peptide is preferentially expressed by hypertrophic chondrocytes of the growth plate and this localization is consistent with a role for this peptide in regulating cartilage mineralization.

### The functional role of MEPE in ATDC5 cells

**Fig. 2:**
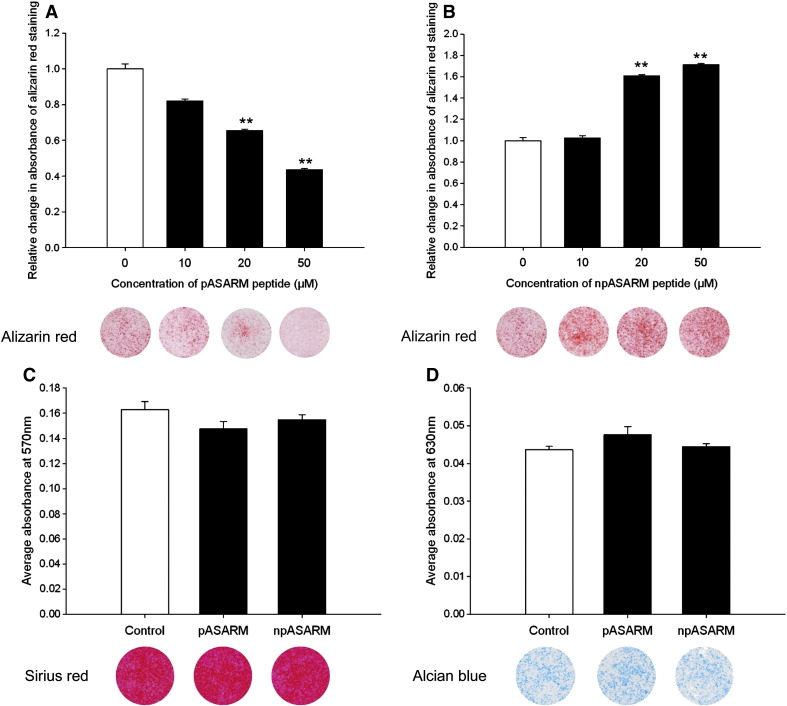
Mineralization of ATDC5 matrix in the presence of (A) pASARM and (B) npASARM peptides was visualised by alizarin red staining (images) and quantified after 15 days of culture. Quantification of sirius red staining (C) and alcian blue staining (D) (images) of ATDC5 cells following 15 days of culture with 20 μM pASARM and npASARM peptides. Data are represented as mean ± SEM of three wells analysed in triplicate. **P < 0.01.

It is known that the C-terminal fragment is the active form of MEPE. This fragment contains the ASARM peptide; thus, we next determined the role of the ASARM peptide in chondrocyte matrix mineralization by examining the mineralization capability of ATDC5 cells in response to MEPE-ASARM peptides. The ATDC5 cell line is a teratocarcinoma derived cell line which has been shown to display the multistep chondrogenic differentiation process, from mesenchymal condensation to matrix mineralization [26,34], at approximately day 15 of culture. The culture method used here did not result in metabolic stress leading to cell death as indicated by assessment of released LDH activity as a percentage of total LDH release (0 mM βGP 33.5% ± 2.5, 10 mM βGP 35.2% ± 0.9, NS). Here we added pASARM and npASARM peptides to ATDC5 cell cultures under calcifying conditions over a 15-day culture period. There was no apparent morphological difference between control and ASARM-treated cells. pASARM peptides inhibited mineralization in a dose-dependent manner as visualised by alizarin red staining and quantified by spectrophotometry (at 20 μM and 50 μM in comparison to control; *P* < 0.01) (Fig. 2A). Interestingly, it was found that npASARM promoted mineralization over the 15-day culture period (at 20 μM and 50 μM in comparison to control; *P* < 0.01) (Fig. 2B). Given that MEPE has been postulated to have direct effects on osteoblast mineralization and not via altered matrix production [14,18], we investigated whether this was the case with ATDC5 cells by examining their ability to produce their collagenous matrix when treated with the MEPE-ASARM peptides. Collagen deposition (Fig. 2C) and glycosaminoglycan production (Fig. 2D), as visualised by sirius red and alcian blue stains, respectively, were unaffected by addition of 20 μM pASARM or npASARM peptide. These data are therefore supportive of a direct role for MEPE-ASARM peptides in chondrocyte matrix mineralization.

**Supplemental Fig. 1:**
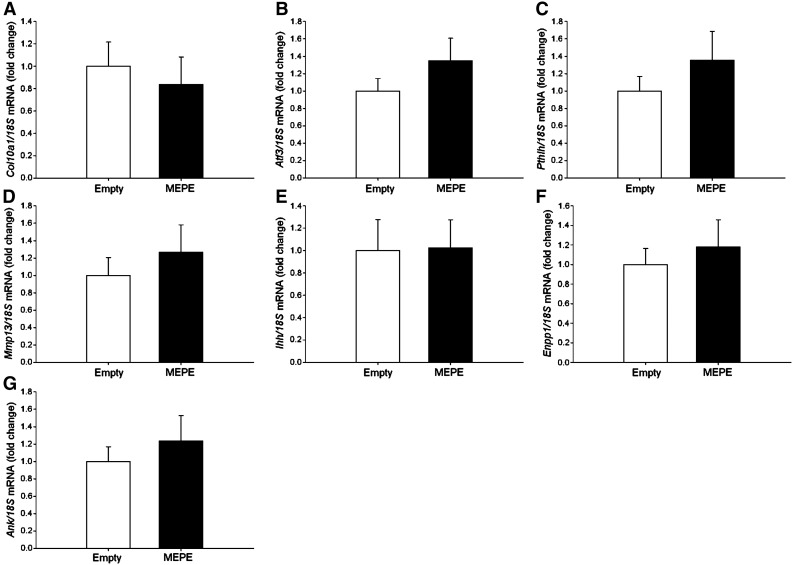
Analysis of mRNA expression in MEPE-overexpressing and empty vector control clones after 15 days of culture. (A) *Col10a1*. (B) *Atf3*. (C) *PthIh*. (D) *Mmp13*. (E) *Ihh*. (F) *Enpp1*. (G) *ank*. Data are represented as mean of 3 clones ± SEM.

**Fig. 3:**
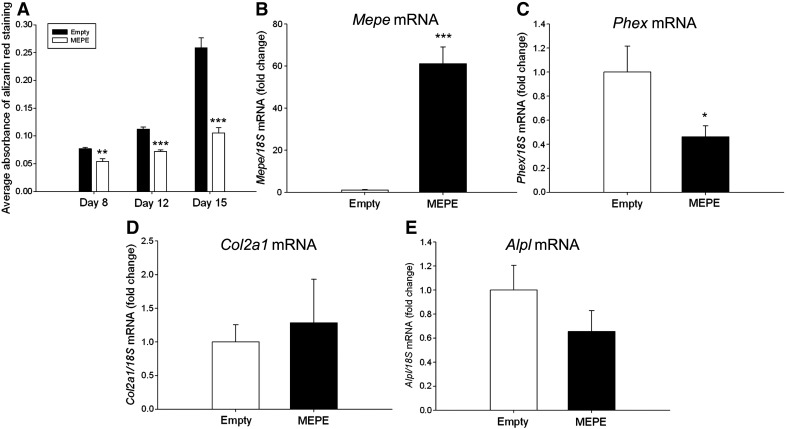
MEPE-overexpressing ATDC5 cells showed inhibited mineralization in comparison to empty vector cells at days 8, 12 and 15 of culture as visualised by alizarin red staining and quantified by spectrophotometry (A). *Mepe* mRNA expression was significantly increased in MEPE-overexpressing ATDC5 clones (B), whilst *Phex* mRNA expression was significantly decreased in comparison to empty vector controls (C). There was no difference in mRNA expression levels of (D) *Col2a1* or (E) *Alpl*. Data are represented as mean of 3 clones ± SEM. *P < 0.05, **P < 0.01, ***P < 0.001.

We next overexpressed MEPE in ATDC5 cells to examine this functional role further. When cultured under calcifying conditions, MEPE-overexpressing cells showed an inhibition of matrix mineralization throughout the culture period as visualised by alizarin red staining and quantified by spectrophotometry (at day 8 in comparison to empty vector control *P* < 0.01, at days 12 and 15 in comparison to empty vector control *P* < 0.001) (Fig. 3A). RT-qPCR amplifications showed that stable individual MEPE-overexpressing ATDC5 cell clones expressed significantly higher *Mepe* mRNA levels than individual empty vector clones (*P* < 0.001) (Fig. 3B). *Phex* mRNA levels were significantly decreased in the MEPE-overexpressing clones in comparison to the empty vector controls (*P* < 0.05) (Fig. 3C). Chondrocyte marker genes of differentiation and mineralization were examined for mRNA expression and no differences were found between the MEPE-overexpressing and the empty vector controls (Fig. 3D and E, Supplemental Fig. S1).

### Phosphorylated MEPE-ASARM peptides inhibit the mineralization capability of E17 metatarsal bones

**Supplemental Fig. 2:**
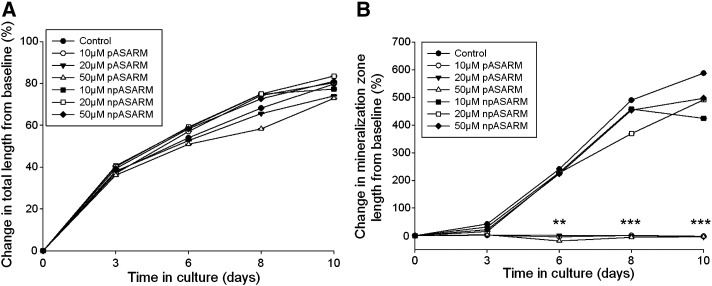
(A) The growth rate of E17 metatarsal bones was not affected by treatment with 10, 20 or 50 μM MEPE-ASARM peptides when cultured for up to 10 days. The percentage change in mineralization zone increased in control and npASARM treated bones, whereas the mineralization zone in bones treated with pASARM peptides did not increase at all during the culture period (B). Data are represented as mean ± SEM of six bones **P < 0.01, ***P < 0.001. Error bars are too small to be visualised.

**Fig. 4:**
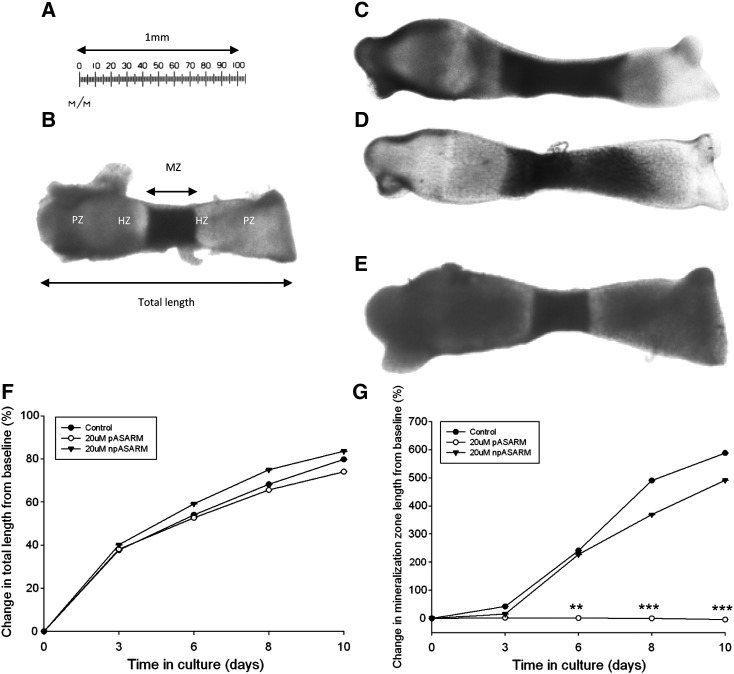
Measurements of digital images of E17 mouse metatarsal bones in culture with clearly delineated mineralizing zones (B–D) were taken using a calibrated ruler (A). Images clearly show the harvesting length (B) with the locations of the proliferating (PZ), hypertrophic (HZ) and mineralizing (MZ) zones, as well as the total length measurement. A control metatarsal bone is illustrated in (C) and bones treated with continuous 20 μM npASARM (D) and pASARM peptides (E) after 10 days of culture. The growth rate of the embryonic metatarsal bones was not affected by treatment with 20 μM MEPE-ASARM peptides (F) when cultured for up to 10 days. There was no significant difference in the percentage change in mineralization length between control and npASARM treated bones, both of which increased over the culture period. However the mineralization zone length in bones treated with pASARM peptides remained the same during the culture period (G). Data are represented as mean ± SEM of six bones. **P < 0.01, ***P < 0.001 in comparison to control bones at equivalent days of culture. Error bars are too small to be visualised.

We next wanted to examine the effects of the MEPE-ASARM peptides on a more physiologically relevant model. Primary chondrocytes provide difficulties when culturing as they tend to dedifferentiate to a fibroblastic-like phenotype during long-term culture [35–38]; thus, we utilized the metatarsal organ culture model. When dissected, E17 mice metatarsals display a central core of mineralized cartilage juxtaposed by a translucent area on both sides representing the hypertrophic chondrocytes [22] (Fig. 4B). These bones were cultured in the presence of varying concentrations of pASARM and npASARM peptides over a 10-day period to examine their effects on longitudinal bone growth and the growth of the central mineralization zone. This preliminary data indicated that MEPE-ASARM peptides inhibit mineralization of metatarsal bones across a range of concentrations (Supplemental Fig. S2). Due to the physiological relevance of 20 μM in XLH patients and *Hyp* mice, this concentration was used throughout these experiments [18]. Bones treated with 20 μM MEPE-ASARM peptides grew in length at the same rate as the control bones (up to 80%) after 7 days in culture (Fig. 4C–F). However, whereas in the control and npASARM treated metatarsals the central mineralization zone increased in length throughout the culture period (increased approximately 5–6 fold from initial lengths, Fig. 4D and E), in the pASARM treated cultures no changes in length were noted (*P* < 0.01 at day 6, *P* < 0.001 at days 8 and 10 in comparison to the control) (Fig. 4C, E and G).

### The effects of the MEPE-ASARM peptides on E15 metatarsal bones

**Fig. 5:**
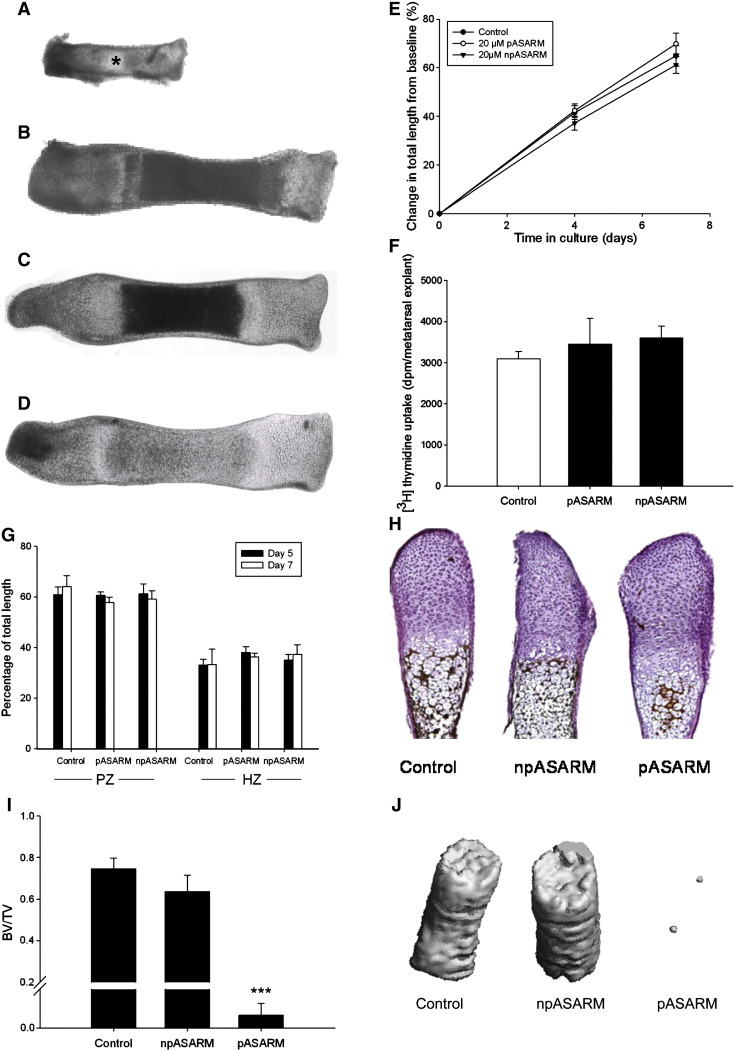
Measurements of digital images of E15 mouse metatarsal bones in culture. At time of harvesting, bones did not have a central mineralization zone as indicated by the asterisk (A). After 7 days in culture, control (B) and npASARM (C) treated bones formed a large mineralization zone; however, this was inhibited in pASARM treated bones (D). All metatarsal bones grew at a similar rate (E) and there was no difference in the proliferation of the chondrocytes within (F). Histological sections (H) showed control and npASARM treated bones to have abundant mineral as indicated by von kossa staining. This was not seen in pASARM treated metatarsals. There was no difference in the widths of the proliferating zone (PZ) and hypertrophic zone (HZ) of chondrocytes between the different groups of metatarsals at either day 5 or day 7 of culture (G). Data are represented as mean ± SEM of six bones. μCT analysis of metatarsal bones treated with npASARM and pASARM peptides. Bones treated with pASARM had a significantly reduced BV/TV in comparison to the control and npASARM treated bones (I). This was clearly visible in the μCT images (J). Data are represented as mean ± SEM of three bones. ***P < 0.001.

To examine this apparent inhibitory effect further, we next determined the effects of the pASARM and npASARM peptides on E15 metatarsal bones. These bones consist of early proliferating chondrocytes (Fig. 5A) and no evidence of a mineralized core. After 7 days in culture, the chondrocytes in the centre of the bone become hypertrophic and mineralize their surrounding matrix as is previously documented [25] (Fig. 5B). This central core of mineralized cartilage formed in control bones and bones treated with 20 μM npASARM peptides (Fig. 5B and C); however, it was minimal in metatarsal bones treated with 20 μM pASARM peptides (Fig. 5D), as seen in the phase contrast images. This was further confirmed by von kossa staining of histological sections for mineralization (Fig. 5H) and by μCT scanning of the metatarsal bones to allow the visualisation of the bones in a 3D context. In comparison to the control and npASARM treated bones, metatarsal bones cultured in the presence of pASARM peptides had a significantly reduced BV/TV (*P* < 0.001) (Fig. 5I), as is clearly visible in the μCT scan images (Fig. 5J). This unequivocally shows the inhibition of mineralization in metatarsal bones by the pASARM peptide. Despite the increase in ATDC5 ECM mineralization upon addition of npASARM peptides, here the mean density of the mineralised bone was unchanged between control and npASARM treated bones (control 163.4 ± 12.1 mg HA/ccm, npASARM 173.2 ± 21.9 mg HA/ccm, not significant).

**Supplemental Fig. 3:**
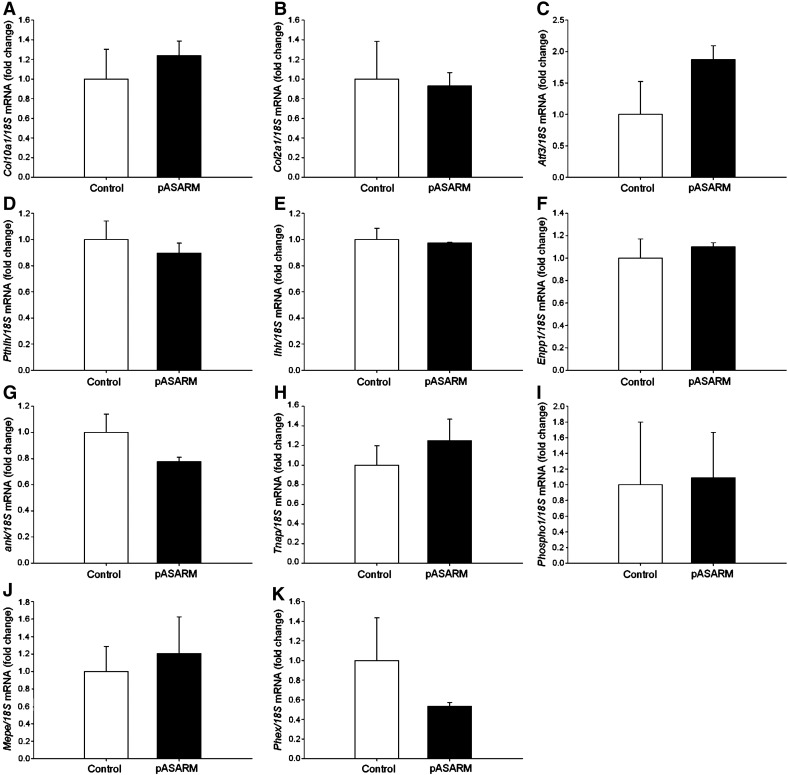
Analysis of mRNA expression in 15-day‐old fetal control and pASARM treated metatarsals at day 5 of culture. (A) *Col10a1*. (B) *Col2a1*. (C) *Atf3*. (D) *PthIh*. (E) *Ihh*. (F) *Enpp1*. (G) *ank*. (H) *Alpl*. (I) *Phospho1*. (J) *Mepe*. (K) *Phex*. Data are represented as mean ± SEM of 3 groups of 4 pooled bones.

**Supplemental Fig. 4:**
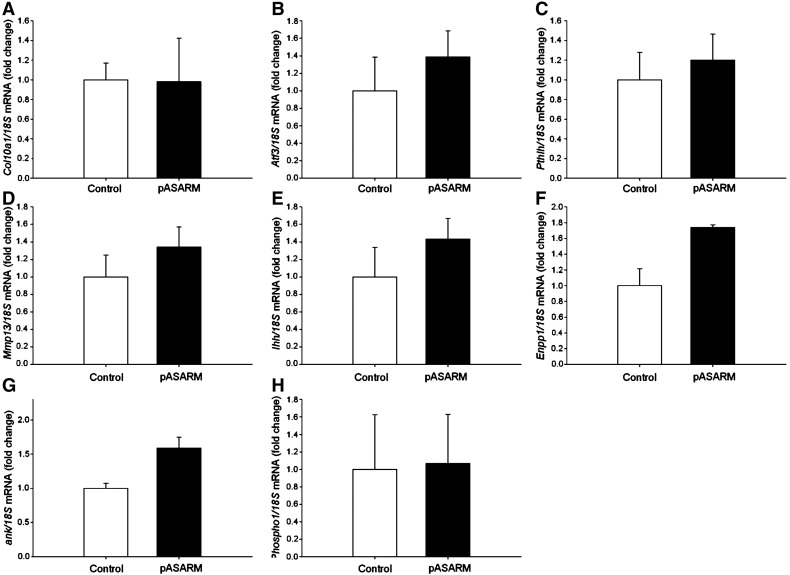
Analysis of mRNA expression in 15-day‐old fetal control and pASARM treated metatarsals at day 7 of culture. (A) *Col10a1*. (B) *Atf3*. (C) *PthIh*. (D) *Mmp13*. (E) *Ihh*. (F) *Enpp1*. (G) *ank*. (H) *Phospho1*. Data are represented as mean ± SEM of 3 groups of 4 pooled bones.

Apart from the inhibition of mineralization by the pASARM peptide, there were no other obvious morphological differences in the development of these bones in comparison to the control bones. All bones grew at the same rate (increased approximately 65% from initial lengths) (Fig. 5E) and by incorporating [^3^H]-thymidine into the bones at the end of the culture period, day 7, it was determined that the proliferation rate of the chondrocytes was unchanged (Fig. 5F). The lengths of the proliferating (PZ) and hypertrophic (HZ) zones of chondrocytes were also measured. The MEPE-ASARM peptides had no effect on the percentage sizes of the maturational zones of the metatarsal bones, or on the cell numbers within the bones (Control: 1139.13 ± 172.01, pASARM: 1594.97 ± 226.9, npASARM 1233.71 ± 126.08). This therefore suggests that the MEPE-ASARM peptides had no effect on the differentiation capability of the metatarsal chondrocytes (Fig. 5G). To examine this further, we looked at mRNA expressions of chondrocyte differentiation markers for which there were no significant differences between the control and pASARM treated bones at days 5 and 7 of culture (Supplemental Fig. S3, Supplemental Fig. S4) as is in concordance with our histological and proliferation data.

**Fig. 6:**
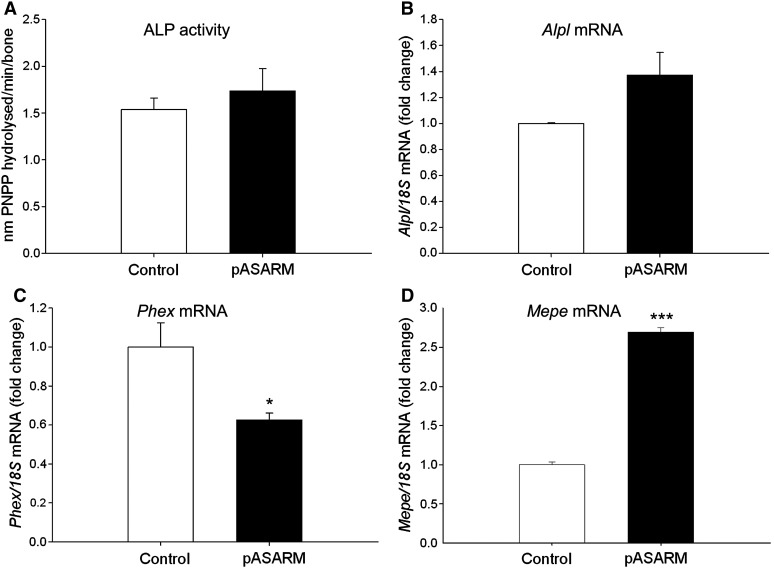
Alkaline phosphatase (ALP) activity was unchanged in metatarsal bones treated with 20 μM pASARM peptides in comparison to control bones (A). *Alpl* mRNA expression was also unchanged (B). *Phex* mRNA expression was significantly decreased in pASARM treated bones (C) whilst *Mepe* mRNA expression was increased (D). Data are represented as mean ± SEM. *P < 0.05 ***P < 0.001 of 3 groups of 4 pooled bones at day 7 of culture.

We also examined the expression and activity of key enzymes associated with cartilage mineralization to establish whether these are involved in the mechanism of inhibition by the pASARM peptides. Interestingly there was no significant difference in the activity of ALP (Fig. 6A), a well recognised regulator of chondrocyte matrix mineralization. This was further confirmed by mRNA expression analysis of *Alpl* by RT-qPCR (Fig. 6B). Analysis of the mRNA expression of other mineralization regulators, *Ank*, *Enpp* and *Phospho1*, also showed no difference between control and treated bones at days 5 and 7 of culture (Supplemental Figs. S3 and S4).

To assess the possible interactions of PHEX with MEPE, we examined mRNA expression of *Phex* and found it to be significantly decreased in the pASARM treated bones compared to the control bones at day 7 of culture (*P* < 0.05) (Fig. 6C). Furthermore, *Mepe* mRNA expression was significantly increased (*P* < 0.001) (Fig. 6D). At day 5 of culture, there was no significant difference in the mRNA expression of *Mepe* or *Phex* (Supplemental Fig. S3).

**Fig. 7:**
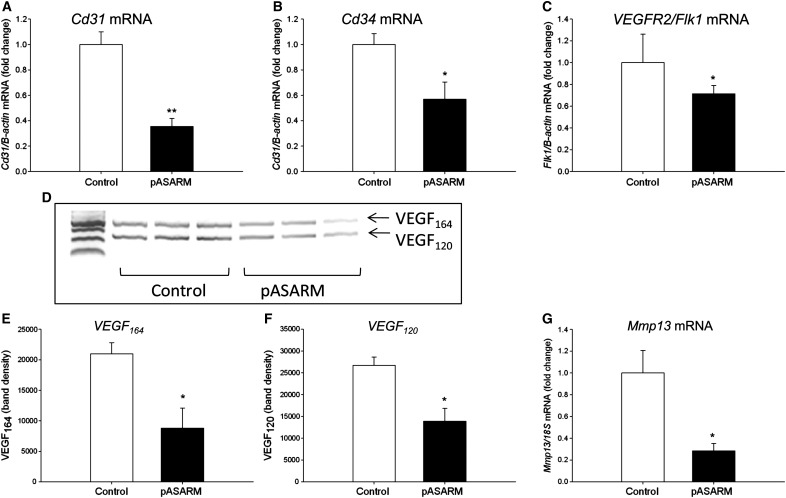
mRNA expression of endothelial cell markers *Cd31* (A), *Cd34* (B), and *VEGFR2*/*Flk1* (C) in control and pASARM treated bones at day 7 of culture. PCR analysis of pro-angiogenic VEGF-A splice variants (D) and densitometry of the VEGF_164_ isoform (E) and the VEGF_120_ isoform (F). mRNA expression of *Mmp13* in control and pASARM treated bones at day 5 of culture (G). Data are represented as mean ± SEM. *P < 0.05, **P < 0.01 of 3 groups of 4 pooled bones. PCR analysis represents replicates of pooled bones at day 7 of culture.

The vascular invasion of the cartilage model via VEGF stimulated angiogenesis is critical for matrix mineralization [39]. Thus, we examined the effects of the pASARM peptide on the mRNA expression of endothelial cell specific markers and VEGF. We found a significant decrease in the expression levels of *Cd31*, *Cd34*, and *VEGFR2*/*Flk1* following 7 days of culture in the presence of 20 μM pASARM compared to controls (*P* < 0.01, *P* < 0.05) (Fig. 7A–C). Furthermore, we also found a concomitant decrease in VEGF isoform expression specifically VEGF_164_ and _120_ (Fig. 7D–F). VEGF_188_ was not detected in either control or treated metatarsals. Matrix metalloproteinase 13 (MMP13), which has been implicated in VEGF-induced angiogenesis [40,41], also had a significantly decreased mRNA expression following 5 days of culture (in pASARM treated bones compared to control; *P* < 0.05) (Fig. 7G). Despite this there was histologically no apparent inhibition of vascularization in the metatarsal bones.

## Discussion

The hypertrophic chondrocytes of the epiphyseal growth plate mineralize their surrounding ECM and facilitate the deposition of HA, a process imperative for longitudinal bone growth. It is widely accepted that ALP, NPP1 and ANK are all central regulators of levels of PP_i_, a mineralization inhibitor, and thus the deposition of HA [42–46]. Recently it has come to light that mechanisms beyond the supply and hydrolysis of PP_i_ also exist to control matrix mineralization. Studies into rare genetic disorders, such as X-linked hypophosphatemic rickets (XLH), have identified a family of proteins, FGF23, PHEX, and MEPE which act through a bone-kidney axis to modulate phosphate homeostasis and thus bone mineralization indirectly [4,47–49]. However, these proteins have been shown to have direct effects on mineralization, independent of the bone-kidney axis [50,51]. Here we provide evidence for MEPE as a novel regulator of growth plate cartilage mineralization.

MEPE is a member of the SIBLING family of proteins and is expressed by mature osteoblasts, osteocytes, odontoblasts and the proximal convoluted tubules of the kidney [12,16,52,53]. It is degraded by cathepsin B to an acidic, negatively charged ASARM peptide which inhibits osteoblast matrix mineralization by directly binding to HA [14,15,18]. Patients with XLH have elevated serum levels of this ASARM peptide as does the mouse model of XLH, the *Hyp* mouse [54]. Further studies of the *Hyp* mouse show severe morphological disruption of the growth plate which can be corrected by the administration of cathepsin inhibitors [16]. This growth plate disruption is also observed in mice overexpressing MEPE [13]. Here we provide evidence of the spatial localization pattern of MEPE and its mRNA in the growth plate; more specifically we have shown it to be predominantly expressed by the terminally differentiated hypertrophic chondrocytes. It is recognised that due to the binding nature of MEPE to HA, EDTA decalcification may in fact provide an underestimation of the total MEPE/ASARM protein produced however the results seen here are consistent with those observed in the MEPE-overexpressing mouse and with a presumed role for MEPE in regulating the fine balance of mineral formation at the growth plate. The localization of cathepsin B at the chondro-osseous junction is in concordance with previous studies detailing the cathepsin B rich septoclast [32,33]. These cells, thought to be of macrophage or osteoclast origin, are postulated to play a key role in the degradation of unmineralized cartilage [33]. It is likely that the cathepsin B provided at the chondro-osseous junction cleaves MEPE at its distal COOH-region to the ASARM peptide which we have shown here to be localised exclusively to the hypertrophic chondrocyte region.

Previous studies have shown the ASARM peptide to inhibit matrix mineralization in in vitro osteoblast cultures [15,18,55]. It is well recognised that the post translational phosphorylation of the MEPE-ASARM peptide is essential for its inhibitory role. Here we utilized the metatarsal organ culture model, a well‐established model of cartilage mineralization and endochondral bone growth. Developmentally in mice by E15, the point at which we use metatarsal bones in these studies, despite a considerable degree of periosteal ossification occurring in the long bones, the metatarsal bones exist as a cartilage model. Here our results unequivocally show that the phosphorylated ASARM peptide (pASARM) has a significant inhibitory role on chondrocyte matrix mineralization. Here we report no difference in the widths of the cartilage zones in the metatarsal bones. A widening of the hypertrophic zone would be expected as seen in hypophosphatemic rickets, and as is observed in the MEPE-overexpressing mouse [13]. This is not surprising though as there was also no difference in the growth potential, chondrocyte proliferation or mRNA expression of chondrocyte differentiation markers, of the treated and untreated bones. This therefore suggests that the MEPE-ASARM peptide has no effect on chondrocyte function per se. Instead it affects chondrocyte matrix mineralization directly, as is in concordance with studies done on bone mineralization [14,18].

It is well recognised that ALP activity is a key regulator of cartilage matrix mineralization. ALP is located to the outer surface of the trilaminar membrane of MVs, which form from the hypertrophic chondrocytes [56]. It is widely accepted that ALP generates P_i_ for HA formation and its lack of activity results in an excess of PP_i_ [57]. The interaction between ALP, PP_i_ and other SIBLING proteins has previously been documented [57,58]. It was therefore postulated that the effects of the pASARM peptide could act through a decrease in ALP activity/expression as has been shown in a previous study of bone mineralization and as is observed in the MEPE‐overexpressing mouse [13,14]. However here we show no effect on ALP activity or expression by the ASARM peptide and as is in concordance with a previous study investigating the role of MEPE in osteoblast mineralization [18]. No effect was also seen on PHOSPHO1 expression, which together with ALP regulates bone and cartilage mineralization suggesting that in the models utilized here, the mechanism of inhibition is not a result of decreased enzyme activity [59,60]. Rather, it is likely that the pASARM peptide exerts its effects through its direct binding to the HA as has previously been suggested.

It has recently been shown that a truncated form of MEPE, which has the ASARM peptide removed, can promote bone mineralization in culture and in mice [61]. Furthermore, a mid-terminal fragment of MEPE has been shown to enhance cell binding and taken together these results highlight the importance of the post translational processing of MEPE in determining its functional role [62]. Here we have shown that the phosphorylation of the ASARM peptide is crucial in determining its functional role. Despite the observed promotion of mineralization by the npASARM peptide in the ATDC5 cultures, this was not corroborated by our metatarsal data. Furthermore in other in vitro studies, it has been shown that the function of the MEPE-ASARM peptide is entirely dependent upon its phosphorylation [14,18,63]. Indeed it is likely that the npASARM peptide does not physiologically exist and is in fact inactive. One can reasonably infer that since the pASARM serine-phosphorylated casein kinase sites are highly conserved across species (including whales, dolphins, primates, rodents, marsupials, elephants, dogs, and cats) and the phosphorylated form is active that there might be a physiological mechanism that plays a role in regulating the ASARM-phosphorylation status [64].

PHEX protects MEPE from cathepsin B cleavage in vitro [17,65]; thus, the inhibition of *Phex* mRNA expression in pASARM treated metatarsal bones and in ATDC5 cells overexpressing MEPE suggests a feedback mechanism by which ASARM peptides can prevent PHEX expression. This, in correlation with an increase in *Mepe* expression seen, would allow the release of ASARM peptides therefore further increasing the inhibition of mineralization. Furthermore, the reduction in *Phex* mRNA expression may be due to the ASARM peptide protecting itself from sequestration and hydrolysis by PHEX, as has previously been suggested [14,18,66]. A decrease in *Phex* mRNA has also been observed in osteoblast cell cultures treated with the pASARM peptide, concomitant with an increase in FGF23 expression [14]. In the MEPE-overexpressing mouse, however, an increase in *Phex* mRNA is observed and this, coupled with the expected hydrolysis of the ASARM peptide, leads to altered MEPE processing and therefore the hyperphosphatemia observed in this mouse model [13]. These data are also in agreement with previous reports showing increased MEPE expression by osteoblasts of HYP mice and this positive regulation of MEPE expression by pASARM may exacerbate the condition [4,10,15,66]. It is reasonable to speculate that physiologically there must be a regulatory mechanism to ensure that there is not an overproduction of ASARM peptides and as such a pathological state. The precise nature of the counter balancing mechanism is presently unknown but as the SIBLING proteins are closely related and it is possible that one of the other members of this family may be responsible.

Key to endochondral ossification is the vascularization of the mineralized matrix [39]. Matrix metalloproteinases (MMPs) proteolytically degrade the mineralized cartilage matrix, facilitating blood vessel penetration into the growth plate and allowing the recruitment of osteoclast precursors and osteoblast progenitors. Pro-angiogenic VEGF is produced by hypertrophic chondrocytes of the growth plate and VEGF_164/188_ deletion from the cartilage of developing mice results in delayed recruitment of blood vessels to the perichondrium along with a delayed invasion of vessels into the primary ossification centre [67]. Here we have shown that the pASARM peptide reduces the levels of endothelial cells present during metatarsal organ culture due to the vessel invasion of the bones at approximately E14– E15. This was associated with reduced VEGF_120/164_ mRNA expression levels. It is entirely possible that the influence of the pASARM peptide on endothelial cell populations is indirect, by impacting hypertrophic chondrocyte VEGF expression. However, any direct effects of the pASARM peptide on endothelial cell function remain uninvestigated. The possible implications of MEPE on bone renal vascularization have recently been described in the MEPE-overexpressing mouse, which in contrast to our studies exhibits defective mineralization associated with increased blood vessels [13]. Similarly, we also found a decrease in *Mmp13* mRNA expression following pASARM treatment which has been implicated in angiogenesis despite there being a lack of impairment of vascularization in the *Mmp13* knockout mouse [40,41,68]. It is likely that in the *Mmp13* knockout and the *Mepe-*overexpressing mice, unknown compensatory mechanisms could exist to allow for effective vascularization of the skeleton. Like MEPE, DMP1, another SIBLING protein, has also been suggested as an inhibitor of VEGF receptor 2 mediated angiogenesis although the precise role of its ASARM peptide in this circumstance has yet to be elucidated [69].

To conclude, our studies detail for the first time the functional role that MEPE and its ASARM peptide have in chondrocyte matrix mineralization. We have shown MEPE to be expressed by growth plate chondrocytes, in particular in the hypertrophic zone of chondrocytes consistent with a role in matrix mineralization. We have shown this role to be dependent upon the extent of the cleavage and subsequent phosphorylation of MEPE, and that mechanisms may exist which positively regulate the further expression of MEPE. Our studies complement previous findings of MEPE and its role in biomineralization; however, much remains to be learnt regarding the in vivo role of MEPE and the ASARM peptide in bone disease.

The following are the supplementary data related to this article.

**Supplemental Table S1:**
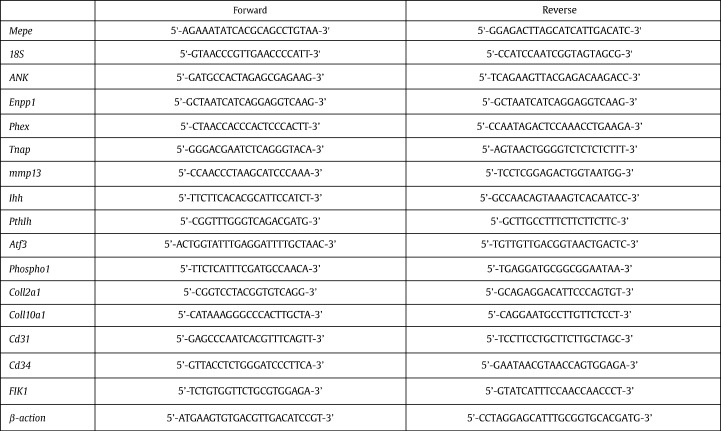
Primers used for RT-qPCR analysis.
